# Exploring the success of an integrated primary care partnership: a longitudinal study of collaboration processes

**DOI:** 10.1186/s12913-014-0634-x

**Published:** 2015-01-22

**Authors:** Pim P Valentijn, Hubertus JM Vrijhoef, Dirk Ruwaard, Antoinette de Bont, Rosa Y Arends, Marc A Bruijnzeels

**Affiliations:** Jan van Es Institute, Netherlands Expert Centre Integrated Primary Care, Randstad 2145-a, 1314 BG Almere, The Netherlands; Scientific Centre for Care and Welfare (Tranzo), Tilburg University, Tilburg, The Netherlands; Saw Swee Hock School of Public Health, National University of Singapore, Singapore, Singapore; Department of Health Services Research, School for Public Health and Primary Care, Faculty of Health, Medicine and Life Sciences, Maastricht University, Maastricht, The Netherlands; Institute of Health Policy and Management, Erasmus University Rotterdam, Rotterdam, The Netherlands; Department of Psychology, Health & Technology, University of Twente, Enschede, The Netherlands

## Abstract

**Background:**

Forming partnerships is a prominent strategy used to promote integrated service delivery across health and social service systems. Evidence about the collaboration process upon which partnerships evolve has rarely been addressed in an integrated-care setting. This study explores the longitudinal relationship of the collaboration process and the influence on the final perceived success of a partnership in such a setting. The collaboration process through which partnerships evolve is based on a conceptual framework which identifies five themes: shared ambition, interests and mutual gains, relationship dynamics, organisational dynamics and process management.

**Methods:**

Fifty-nine out of 69 partnerships from a national programme in the Netherlands participated in this survey study. At baseline, 338 steering committee members responded, and they returned 320 questionnaires at follow-up. Multiple-regression-analyses were conducted to explore the relationship between the baseline as well as the change in the collaboration process and the final success of the partnerships.

**Results:**

Mutual gains and process management were the most significant baseline predictors for the final success of the partnership. A positive change in the relationship dynamics had a significant effect on the final success of a partnership.

**Conclusions:**

Insight into the collaboration process of integrated primary care partnerships offers a potentially powerful way of predicting their success. Our findings underscore the importance of monitoring the collaboration process during the development of the partnerships in order to achieve their full collaborative advantage.

**Electronic supplementary material:**

The online version of this article (doi:10.1186/s12913-014-0634-x) contains supplementary material, which is available to authorized users.

## Background

Integrated-care approaches are increasingly being promoted in order to respond to the challenges of the health care systems in high-income countries. Such challenges include reducing costs, improving quality of care and generating better patient outcomes [1-3]. Primary care, considered the cornerstone of these health systems, has proven to be essential for achieving desired health outcomes and limiting costs [3-5]. Primary care provides patients their first contact with professional health care, facilitates access to other health and social services and coordinates care for those with complex needs [5,6]. In this study, we refer to *integrated primary care* as settings in which a network of multiple professionals and organisations across the health and social care system provide accessible, comprehensive and coordinated services to a population in a community. A key component of integrated service delivery is the collaboration between the different actors involved [7]. Such collaborative partnerships are widely used as a means to provide integrated health care services [8-11]. In this study, the term *partnership* refers to a setting that includes inter-sectorial collaboration as well as inter-organisational and inter-professional collaboration across a network of multiple organisations and professionals [8-11].

The collaboration processes through which partnerships evolve and are sustained have rarely been addressed empirically [12-16]. There is considerable uncertainty surrounding whether and under what conditions all actors (e.g. health care professionals, managers, and policymakers) involved in the partnership will collaborate [17]. This knowledge is important, as collaborative partnerships are often described as time-consuming, resource intensive, and fraught with challenges [18-20]. Especially in the health and social care systems, partnerships tend to have a high and often early failure rate [18]. There is also a lack of empirical evidence showing how the collaboration process influences the success of a partnership over time [21].

Bell, Kaats and Opheij (2013) [22] provided a conceptual framework that consists of five different themes in order to evaluate the collaboration processes of a partnership: 1. Shared ambition (shared commitment of the involved partners), 2. Mutual gains (understanding the various interests of the involved partners), 3. Relationship dynamics (relational capital among the partners), 4. Organisation dynamics (governance arrangements among the partners), and 5. Process management (process steering among the partners). The framework is grounded on a solid base of literature in which the individual themes have been described by various authors. For example, developing a clearly stated shared ambition (e.g. vision and mission) has been emphasized in the literature as an essential aspect of a successful partnership [16,23,24]. Closely related to the shared ambition theme is the mutual gains approach, which refers to the dialogue about the underlying interests of the partners to provide an ideal win-win solution. Numerous scholars [22,25-27] have argued that the mutual gains approach is an essential aspect of developing a sustainable partnership. Another important aspect in the current literature is the relational capital among partners, defined as relational dynamics [16,28-30]. Various researchers [29,31,32] have argued that close interpersonal ties between the partners can act as an effective mechanism to build mutual trust and respect within a partnership. Alliance literature also suggests that formal governance mechanisms, defined as organisational dynamics, are also essential to developing trust and commitment within a partnership [16,33-36]. Finally, a large body of literature has focused on the importance of process management in order to facilitate the complex and delicate nature of forging a collaborative partnership [19,22,37].

**Figure 1:**
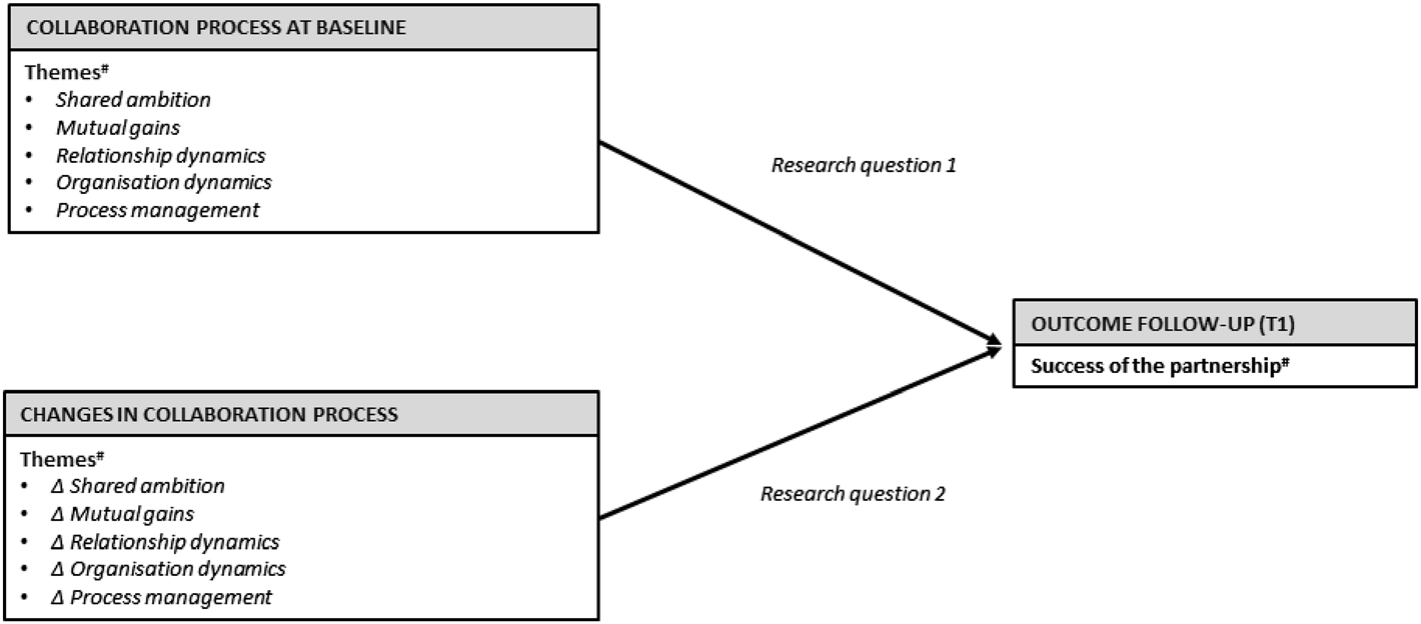
**Hypothesised relations between the collaboration process and the success of the partnership.** ^#^Description and operationalization of the collaboration process themes and success of the partnership are described in the “Methods” section.

Although extensive literature has suggested the importance of the five themes of Bell et al. [22], empirical evidence on the impact of these themes on the success of a partnership over time is limited. By developing an understanding of how the collaboration process can successfully be managed, partners can better know in advance whether the partnership will achieve the desired “collaborative advantage” [38]. The aim of this paper is to explore the relationship between the collaboration process and the perceived success of a partnership. This paper aims to contribute to an understanding of how partnerships can successfully be established and maintained. Given the non-linear, continual change in development of a partnership [22,39], it seems reasonable to evaluate the collaboration process themes at the start and during the partnership, in order to understand how these themes shape its final success. Therefore, we hypothesised that the perceived degree of success of a partnership is influenced by the presence at baseline of the collaboration process themes and their transformation over time. Specifically, this leads to the following research questions: *1) To what extent do the five collaboration process themes at baseline influence the final success of a partnership? 2) To what extent do changes in the collaboration process themes influence the final success of a partnership?* The different themes and their assumed relationships to the perceived success of a partnership are illustrated in our analytical framework (see Figure 1).

## Methods

### Study design and setting

The present study was a longitudinal study conducted among partnerships enrolled in the national integrated primary care programme *Op één lijn* in The Netherlands (translated as “Primary Focus”) [40]. As an initiative from the Ministry of Health, the programme aimed to stimulate integration through partnerships among local health and social services. Existing and new partnerships were invited to submit a grant application for the development and strengthening of their integrated primary care approach. The following criteria were used by the Netherlands Organization for Health Research and Development (ZonMw) to select and fund 69 eligible partnerships: 1) The subject of the partnership is centred on organisational (re)development aiming towards local multidisciplinary collaboration. The partnership needs to be focused on organisational advancement and processes, not on the organisation of patient care itself; 2) The organisational development aims to provide better quality, accessibility, service, efficiency and/or transparency of care; 3) The partnership focuses on local health and/or social service delivery (in a neighbourhood, village or region); 4) The project team of the partnership is multidisciplinary; 5) The partnership aims to create a sustainable organisational structure after the programme has been completed; 6) Patients or patients’ representatives are involved in the partnership; 7) The partnership provides new knowledge about organisational structures and developments in local health care.

Grant applications were assessed for their relevance and quality using the standardised assessment procedure of the ZonMw [41].

As part of the programme, the selected partnerships participated in a longitudinal study from 2010 to 2013. For our evaluation, we used data that was collected at the start (T0) and the end (T1) of the funding period of each partnership. The average funding period of the partnerships was 22.9 months (SD: 7.5, range 5–36) and the average time between the program measurement points was 19.5 months (SD: 7.3, range: 6–38). Fifty-eight out of the 69 partnerships (84%) already existed before the start of the program and were operational (e.g. implementing shared agreements) at T0 of the program. To be included in the analyses, partnerships had to meet the following criteria: 1) form an inter/intra-sectorial, inter/intra-organisational and/or inter/intra-professional collaboration among different professionals and/or organisations, 2) provide data on T0 and T1 of the programme measurement points. Based on these two criteria, 59 partnerships out of 69 were considered to be eligible for this study. Ethical approval was not required under Dutch law, as no patients were involved in this study.

### Data collection procedure

The collaboration process and perceived final success was examined at the strategic level of the partnership. A questionnaire was sent by e-mail at T0 and T1 to all active steering committee members of the partnerships as identified in the original grant application. The mailing list was verified by the coordinator of each partnership. An active steering committee member was defined as any partner who was involved in the administration and strategic decision making processes in order to realise the collaborative objective of the partnership. In order to maximize the response rate, the partnership coordinator was asked to inform the steering committee members about the purpose of the study and the questionnaire. Furthermore, forced answering (e.g. which required respondents to enter a response before they are allowed to proceed to the next survey question) was used to prevent missing answers. An e-mail reminder was sent after one week to non-respondents. Additional data about the characteristics of the partnership were collected by a semi-structured interview with the partnership coordinator at T0 of the programme. For details about these semi-structured interviews, see “Additional file 1”.

### Measurements

A questionnaire was developed to measure the five collaboration themes, the perceived degree of success of the partnership and to collect descriptive information about the partnerships. The primary outcome measurement was the degree of success of the partnership as perceived by the steering committee members of each of the partnerships. Steering committee members were asked to rate the overall success of their partnership at the end of the evaluation programme on a scale from zero (very bad) to ten (excellent). The five collaboration themes were assessed at T0 and T1. Respondents were asked to indicate the extent to which he/she (dis)agreed with a given statement on a 4-point Likert-scale ranging from 1 (not at all) to 4 (totally). Details of the individual items of shared ambition, mutual gains, relationship dynamics, organisational dynamics and process management are provided in Table 1. In addition, the validity of the questionnaire was assessed during the current study.

**Table 1:** **Characteristics of the variables at baseline (T0) and follow-up (T1)**

**Variable**	**Items**	**Range**	**Cronbach’s alpha**	**Baseline (T0)**		**Follow-up (T1)**		**∆ (T1-T0/T0)**	
				**Mean**	**SD**	**Mean**	**SD**	**Mean**	**SD**
*Outcome*									
Perceived success of the partnership	1	0-10	NA	NA	NA	7.34	0.80	NA	NA
*Collaboration process themes*									
Shared ambition*	4	1-4	0.78	3.49	0.27	3.49	0.27	0.01	0.10
Mutual gains**	4	1-4	0.82	3.04	0.34	3.02	0.39	0.00	0.12
Relationship dynamics***	4	1-4	0.73	3.20	0.26	3.27	0.36	0.03	0.11
Organisation dynamics****	6	1-4	0.86	3.02	0.28	3.08	0.37	0.02	0.14
Process management*****	4	1-4	0.80	3.04	0.29	3.11	0.36	0.03	0.13

NA (not assessed).

*Items: a) Is the ambition shared among the partners. b) is the ambition attractive for the partners. c) is the ambition aligned with the collaboration strategy of each partner. and d) does the ambition have a personal significance for the key players in the partnership? Single-factor solution with factor loadings ranging from: 0.671 to 0.887.

**Items: a) Do the partners have sincere interest in one another’s interests. b) do the partners have a dialogue about one another’s interests. c) are the partners willing to negotiate with one another. and d) does the partnership create value for each of the partners? Single-factor solution with factor loadings ranging from: 0.694 to 0.877.

***Items: a) Do the partners have the personal ability to connect. b) does the group processes consolidate the partnership. c) do the partners trust one another. d) is leadership being demonstrated. and e) is leadership being granted? Two-factor solution indicated that item e) did not demonstrate salient factor loading (i.e. > .40). After excluding item e) a single-factor solution with factor loadings ranging from 0.647 to 0.811.

****Items: a) Is the structure of the partnership aligned with the partners’ objective(s). b) is the direction of the partnership aligned with the partners’ objective(s). c) can the partnership count on the support of the management/professionals and stakeholders. d) are the agreements of the partnership clear. e) are the agreements being fulfilled by the partners. and f) does the partnership realize the proposed objective(s)? Single-factor solution with factor loadings ranging from: 0.669 to 0.841.

*****Items: a) Is there a thorough phasing for the planning of the partnership. b) is the shared ambition of the partnership being realised. c) is the attention of the partners balanced between the content and process of the partnership. d) are the roles clearly divided within the partnership. and e) is the collaboration process clearly directed? Two-factor solution indicated that item b) did not demonstrate salient factor loading (i.e. > .30). After excluding item b). a single-factor solution with factor loadings ranging from 0.683 to 0.846.

### Data analyses

Individual responses were aggregated to the partnership level, as the partnership level was the primary unit of analyses. Respondents with more than 30% of missing answers on the collaboration theme items were excluded, as they had stopped their responses on the questionnaire prematurely. Then, the within-partnership variance was examined in relation to the between-partnership variance by using a one-way analysis of variance (ANOVA). The ANOVA test was conducted to determine if it was justifiable to aggregate the individual responses to the partnership level [18,42]. Partnership-level scores were obtained by calculating the scale score for each respondent and then taking the average score of all the respondents within a partnership [18]. Next, the validity and reliability of the collaboration theme subscales at baseline were tested at the partnership level. To test the construct validity, we performed an exploratory principal-component factor analyses with varimax rotation on all of the five subscales [18]. A factor loadings threshold of > .40 was applied to identify items that cluster together [43]. In addition, a Cronbach alpha of > .70 was used as threshold for the reliability of each subscale [43]. Changes in collaboration process were calculated as follows: (scores on T1 – scores on T0)/scores on T0.

Bivariate relationships between the variables were estimated using Pearson correlation coefficients (*r*).

Multiple regression analyses using forced entry were conducted to answer both research questions. Separate analyses were conducted as the sample size limited the number of independent variables that could be included in the model. The variance inflation factor (VIF) was assessed with a threshold of ≤ 10 for acceptable collinearity [43]. To answer the first research question, the association between the collaboration process at baseline and perceived success was examined. A separate regression analysis was conducted to examine the second research question regarding the change in collaboration process. The significant baseline and change variables of the previous analyses were used in a final regression analysis.

Because of the exploratory nature of this study, *p*-values between .05 and .10 were considered suggestive for an association, and correlations with a *p*-value less than .05 were considered as statistically significant [18]. Data analyses were performed using SPSS version 21 for Windows (IBM Statistics).

## Results

### Sample characteristics

Fifty-nine out of the 69 enrolled partnerships (86%) met the criteria for inclusion. Table 2 shows the general characteristics of the 59 partnerships at baseline. The overall individual response across these partnerships was 75% (338 out of 450 questionnaires) at T0, and 75% (320 out of 426 questionnaires) at T1. Seventeen respondents at T0 and 16 respondents at T1 missed > 30% of the collaboration theme items and were excluded, resulting in 321 respondents at T0 and 304 at T1.

**Table 2:** **General characteristics of the 59 partnerships**

*Funding configuration*	
Funding period (months), mean (SD), range	22.9 (7.5), 5-36
Funding (€), mean (SD), range	97.634 (45.846), 32.930-294.100
*Scope and objective*	
Geographic scope, n (%)	
Local community level	46 (78.0)
Regional province level	13 (22.0)
Objective, n (%)	
Chronic care	11 (18.6)
Elderly	10 (16.9)
Local collaboration	17 (28.8)
Integrating health and social care	14 (23.7)
Other	7 (11.9)
*Organisational configuration*	
Prior history of collaboration	
Yes	50 (84.7)
No	9 (15.3)
Own investment, n (%)	
Yes	45 (76.3)
No	14 (23.7)
Legally formalised, n (%)	
Yes	7 (11.9)
No	52 (88.1)

### Preliminary analyses

The within-partnership variance was significantly less (p ≤ .01) in relation to the between-partnership variance for the collaboration process variables and the success of the partnership. These findings suggest that the mean of the individual responses for each scale within a partnership is a good approximation of the partnership as a whole [18,42].

Exploratory principal components factor analyses with varimax rotation showed that the shared ambition, mutual gains and organisational dynamics resulted in a one-factor solution (see Table 1 notes). Initially, the relationship dynamics and process management scale resulted in a two-factor solution, and in both scales, one item did not demonstrate salient factor loading (i.e. > .40). These items (item e for relationship dynamics and item b for process management) were removed from the two scales, resulting in a satisfactory one-factor solution for both cases. In addition, the reliabilities of the scales were more than adequate, with Cronbach’s alphas ranging from .86 for the organisational dynamic scale and .73 for the relationship dynamic scale. The results of the factor and internal reliability analyses as well as the descriptive statistics for all scales at T0 and T1 can be found in Table 1.

### Correlations

Moderate to strong correlations were found between the collaboration process variables at T0 which were all statistically significant (*p* < .01) (Table 3, rows 1–5). In addition, correlations between the change in collaboration process variables ranged from moderate to strong and were all statistically significant (*p* < .01, rows 6–10). Finally, statistically significant relations (*p* < .01) were found for the variables of mutual gains, relationship dynamics, organisational dynamics and process management at T0 and T1 with the perceived success of the partnership (Table 3, row 11).

**Table 3:** **Pearson correlations for the study variables**

**Variable**	**(1)**	**(2)**	**(3)**	**(4)**	**(5)**	**(6)**	**(7)**	**(8)**	**(9)**	**(10)**	**(11)**
1. Shared ambition	─										
2. Mutual gains	0.68**	─									
3. Relationship dynamics	0.71**	0.69^**^	─								
4. Organisation dynamics	0.67**	0.67^**^	0.80^**^	─							
5. Process management	0.54**	0.55^**^	0.70^**^	0.84^**^	─						
6. ∆ Shared ambition	−0.63**	−0.23	−0.37^**^	−0.32*	−0.26^*^	─					
7. ∆ Mutual gains	−0.39**	−0.37^**^	−0.30^*^	−0.26^*^	−0.07	0.63^**^	─				
8. ∆ Relationship dynamics	−0.30^*^	−0.15	−0.36^**^	−0.22	−0.08	0.61^**^	0.75^**^	─			
9. ∆ Organisation dynamics	−0.35^**^	−0.12	−0.32^*^	−0.42^**^	−0.26	0.71^**^	0.75^**^	0.78^**^	─		
10. ∆ Process management	−0.25	−0.00	−0.28^*^	−0.31^*^	−0.39^**^	0.62^**^	0.50^**^	0.58^**^	0.78^**^	─	
11. Perceived success of the partnership	0.27^*^	0.42^**^	0.33^**^	0.40^**^	0.46^**^	0.32^*^	0.54^**^	0.62^**^	0.55^**^	0.36^**^	─

*p < .05. **p < .01.

∆ change in collaboration process = (T1 score – T0 score)/T0 score.

### Baseline collaboration process

The regression analysis showed the results obtained in response to the two main research questions (Table 4). In order to answer the first research question, Model 1 examined the baseline collaboration process variables that were associated with the perceived success of the partnership at T1. The baseline collaboration process explained 27% of the variance in partnership success. Only mutual gains (β = .36, *p* < .10) and process management (β = .44, *p* < .10) were predictors for the final success of the partnership. None of the other baseline collaboration process variables had a predictive value for the perceived success.

**Table 4:** **Regression analysis predicting the perceived success of a partnership by baseline and change in collaboration process (N = 59)**

**Variable**	**Standardised beta coefficient (β)**	***p*** **-value**
Model 1: Collaboration process at baseline		
Shared ambition	−0.10	0.59
Mutual gains	0.36	0.05*
Relationship dynamics	−0.08	0.72
Organisation dynamics	−0.08	0.76
Process management	0.44	0.05*
Model 2: Change in collaboration process		
∆ Shared ambition	−0.22	0.16
∆ Mutual gains	0.16	0.39
∆ Relationship dynamics	0.45	0.02*
∆ Organisation dynamics	0.30	0.23
∆ Process management	−0.77	0.66
Model 3: Combined		
Mutual gains	0.35	0.00***
Process management	0.32	0.00***
∆ Relationship dynamics	0.70	0.00***

∆ change in collaboration process = (T1 score - T0 score)/T0 score.

Model 1: R^2^ = .27**. Model 2: R^2^ = .43*** and Model 3: R2 = .72***.

*p ≤ .05. **p < .01. ***p < .001.

### Change in collaboration process

To answer the second research question, Model 2, as shown in Table 4, was examined for association between the change variables of the collaboration process and the final perceived success of the partnership. Together the change variables of the collaboration process explained 43% of the variance in success, and change in relationship dynamics was found to be the greatest predictor of success (β = .45, *p* < .05). None of the other change variables of the collaboration process had a predictive value for the final perceived success.

### Combined model

Model 3 of Table 4 identified the association between the significant baseline and changed variables of Models 1 and 2 along with the perceived success of the partnership. Together, these variables explained 72% of the variance in partnership success. Mutual gains (β = .35, *p* < .001) and process management (β = .32, *p* < .001) at baseline and the change in relationship dynamics over time (β = .70, *p* < .001) were predictors for the final success of the partnership.

## Discussion

The aim of this study was to gain a better understanding of the collaboration processes and their relation to the perceived success of a partnership. Partnerships that were more positive about mutual gains and process management at baseline had a significant higher level of perceived success (research question 1). Additionally, partnerships that demonstrated an increase in relationship during the collaboration process also had higher levels of perceived success (research question 2).

### Contribution of research findings

To the best of our knowledge, this research is among the first empirical studies to explore how changes in the collaboration process influence the final success of a partnership in an integrated primary care setting. An intriguing finding was that the mutual gains approach at baseline, e.g. being explicit and voicing the interests of the partners, was one of the preconditions related to the success of a partnership. Although the mutual gains approach is considered as an ongoing aspect of the successful functioning of a partnership [22,25-27], mutual gains did not change in the partnerships during our study. Process steering at the start of a partnership, defined as process management, played another crucial role in explaining the final success of a partnership. When comparing the collaboration process themes at the start with the change of collaboration process over time, only relationship dynamics appeared to have a significant effect on the final success of the partnership. This result highlights the importance of building relational capital during the developmental phase of a partnership and is consistent with previous research [16,28,29].

We found no association between higher scores of shared ambition (e.g. vision and mission of the partnership) and the perceived success of a partnership, even though a clear vision and mission is widely regarded as an essential condition for a successful partnership [16,23]. This might be explained by the evolution of the partnerships included in this study. One explanation could be that the partnerships had already developed and sustained a shared ambition at the start of the study, partly as a result of applying for the grant from the funding agency. Likewise, the majority of the partnerships were already formed before the start of the program.

Furthermore, our findings show that organisation dynamics did not appear to have any importance on the final success of a partnership. We found this result surprising given the focus of the “Primary Focus” programme on the organisational arrangements within the partnerships (see Methods section). The need for effective organisational arrangements is suggested in various academic fields (e.g. economics, business administration, management, and public health sciences) [16,33-36]. Existing literature also suggests that both trust-based (relationship dynamics) and control-based (organisational dynamics) governance mechanisms play a crucial role in partnership development [34,35]. Given the fact that an increase in relationship dynamics during the programme had a significant effect on the perceived success, this may indicate that trust-based governance mechanisms are of more importance in the development of integrated primary care projects.

### Implications for practice and research

Our findings can help to improve the formation and development of a partnership, as many partnerships struggle to realise their collaborative advantage [16,18-20,38]. The strength of this study is the longitudinal design, which allowed studying a more causal relationship between collaboration process and the perceived success of a partnership. This knowledge is a vital step to understanding and improving the collaborative advantage of integrated care approaches. Another positive point of this study was the relatively high response rates (75%) at both time points. The forced answering method and the cooperation with the project coordinators during the data collection process likely contributed to the high response rates.

The study has also some limitations. Although the included partnerships in this study varied in their duration, scope, objectives and size, they constituted a convenience sample. Due to the potential bias of the selected participants, caution should be taken when generalizing the results of this study. For example, positive results are likely to be overrepresented. Through the selection process of the funding agency, more successful partnerships could have been selected. In particular, the dependency of the partnerships on funding could have resulted in more positive reporting by the steering group members in order to be perceived more favourably for future funding. Moreover, the use of self-reported data always involves risks of social desirability and differences in recall [44]. Furthermore, this study represents the managerial perspective of the steering group members within a partnership. Therefore, the results cannot be generalized without reservations to reflect the perspectives of all the actors (e.g. clients, professionals or policymakers) involved in an integrated primary care setting [2,7]. In addition, to our knowledge, this is one of the few studies that used a survey to study the collaboration process, with little empirical precedent to develop most of the measures that were used. Although the construct validity was assessed at baseline, the reliability of the scales over time (i.e. test-retest reliability) was not assessed. Therefore, there is scope to improve and refine some of the measures used in this study.

Future research should focus on the development of outcome measures that represent the different perspectives of all actors (e.g. policymakers, managers, health care professionals and patients), and can be used as a proxy for partnership performance. Furthermore, it would be useful to develop additional measures using objective data (e.g. meeting hours along with methods and frequency of contacts among partners) and to examine how they relate to corresponding self-report measures. Future research should also examine how the theoretical relationships considered in this study are related to the actual impact of a partnership on health and cost-related outcomes [3].

## Conclusion

The findings of this study allow us to better understand the underlying collaboration process and offer a potentially powerful method to predict the success of an integrated primary care partnership. Our results indicate that managing a successful partnership within an integrated primary care context explicitly requires partners’ interests and process management at the start, and, subsequently, the building of relational capital throughout the collaboration process. While our findings do not guarantee the success of a partnership, our results do underscore the importance of monitoring the collaboration process which underlies the development of partnerships in order to achieve their full collaborative advantage.

## Additional file

Additional file 1:
**Semi-structured interview guide.**
