# Supplementary material for: Exploring the success of an integrated primary care partnership: a longitudinal study of collaboration processes
Source: BMC Health Serv Res. 2015 Jan 22;15:32. doi: 10.1186/s12913-014-0634-x (PMC4310187; doi:10.1186/s12913-014-0634-x)
Supplement: Additional file 1: — Semi-structured interview guide. [file 12913_2014_634_MOESM1_ESM.docx]

**Additional files**

**Additional file 1 – Semi-structured interview guide**

The following elements were addressed during the interview with the project coordinator at T0.

**General objectives of the partnership**

- What are the general objectives of the partnership?
- What are the intended outcomes of the partnership?
- What is the geographical scope of the partnership (e.g. local. regional or national)?

**The organisational (financial and legislative) structure of the partnership**

- Is the partnership legally formalised (e.g. foundation. association. private company)?
- Do the participating organisations invest in the partnership?
- How do the participating organisations invest in the partnership?

**Process management activities**

- Did the participating organisations worked together before?
- Could you describe the prior collaboration activities?
